# Route towards extreme optical pulsation in linear cavity ultrafast fibre lasers

**DOI:** 10.1038/s41598-018-31725-7

**Published:** 2018-09-06

**Authors:** Ahmet E. Akosman, Michelle Y. Sander

**Affiliations:** 1 Department of Electrical and Computer Engineering, Boston University, 8 St. Mary’s Street, Boston, MA 02115 USA; 2 Photonics Center, Boston University, 8 St. Mary’s Street, Boston, MA 02115 USA; 3 Division of Materials Science and Engineering, Boston University, 15 St. Mary’s Street, Brookline, MA 02446 USA

## Abstract

Pathways towards the generation of extreme optical pulsation in a chaotic transition regime in a linear fibre laser cavity configuration are presented. In a thulium mode-locked fibre laser, extreme events that can be controllably induced by manipulating the cavity birefringence for pulse energies exceeding the single soliton pulse operating regime are studied in detail for the first time. While a solitonic pulsation structure at the fundamental repetition rate is maintained, additional energy is shed in a chaotic manner, leading to broader spectral generation and shorter pulse durations whose behaviour deviates significantly from a classical statistical distribution. These pulses display markedly different characteristics from any previously reported extreme events in fibre lasers associated with multiple solitons and pulse bunching, thus presenting a novel observation of extreme pulsation. Detailed noise studies indicate that significant enhancement of relaxation oscillations, modulation instability and the interplay with reabsorption mechanisms contribute in this transient chaotic regime. The extreme pulsation generated in a compact fibre laser without any additional nonlinear attractors can provide an attractive platform to accelerate the exploration of the underlying physics of the chaos observed in mode-locked laser systems and can lead to novel fibre laser cavity designs.

## Introduction

Passively mode-locked fibre lasers have attracted significant scientific attention during the past decades as a compact and highly stable platform to explore novel ultrafast phenomena^1–3^. Since passively mode-locked fibre lasers employ a rich set of optical effects such as gain and loss dynamics, group velocity dispersion and nonlinearities during their operation, numerous unprecedented ultrafast pulsation schemes have been explored beyond the conventional stable solitonic pulses with equal intensities and a uniform temporal distribution. In these regimes, the design of the laser cavity and its characteristic parameters can influence the stability and instability regions of operation significantly. By manipulating the cavity birefringence in fibre lasers, new pulse forms beyond conventional scalar solitons have been induced, including vector solitons^4–6^ that, for example, can undergo cross-polarization coupling effects^7^ and take various forms of coupled bright and dark soliton states^8,9^. Disordered ultrafast pulses and pulse trains have been demonstrated in studies including polarization disorder^10^, stochastic pulsation^11^, noise like pulses^12–14^, rogue waves^15^, and soliton molecules^16,17^, rains^18,19^, bunching^20,21^ and explosions^22^. The increased disorder is caused by operating the mode-locked fibre laser in a dissipative regime where the pulses undergo significant amplitude shaping before their final shape is obtained^23^. Noise-like pulsation^12,13^ can be achieved through a strong manipulation of the cavity loss profile, resulting in quasi-stable pulses with a wide pedestal in the autocorrelation traces with a coherent spike. The encountered enhanced nonlinearities in these operating regimes generally lead to a wider optical spectrum bandwidth. Soliton condensation, including the generation of soliton molecules^16,17^, rains^18,19^, bunching^20,21^ and explosions^22^, generally emerge from extensive noise background combined with pronounced nonlinearities and a modified overall cavity loss profile. These condensed states generally lead to the generation of solitons composed of either paired or aggregated pulses. The induced changes in the intrapulse shaping can be imprinted on the optical spectrum as fringes or additional side peaks, and are characterized by a deviation from the conventional sech or parabolic spectral profile, dependent on the overall net cavity group delay dispersion. In addition, their autocorrelation traces usually feature wider pedestals or wings.

The level of disorder in the pulse train can be further elevated to reach chaotic pulsation with similarities to optical rogue waves (RWs). RW were first observed and characterized in oceanography as giant ocean waves exhibiting at least two times higher amplitudes than the surrounding average waves^24^. The occurrence of extreme events in a stable solution set is well described in dynamical-statistical theories including the effects of modulational instabilities^25^, linear space-time focusing^26^, optical feedback leading to bifurcations^27^ and weak nonlinear attractors^28^. As optical fibres offer an attractive test beds for enhanced nonlinear effects, RW behaviour in optical signals has been widely studied^29–33^. Optical RW generation has also been demonstrated in nonlinear optical cavities^34^ and Raman fibre amplifiers^35^. Optical RWs have also been observed in passively mode-locked fibre lasers, which all featured ring cavity configurations, generated by different mechanisms including soliton explosions and collisions^36,37^, chaotic pulse bunching^21^, enhanced Raman dynamics^33^ and spectral filtering^38^. Optical RWs can form an interesting operational regime to better understand the complex and rich nonlinear dynamics of mode-locked lasers and enhance the understanding of performance stability for new laser cavity designs.

While seminal research on optical RW generation has been presented in erbium^21,36,37^ and ytterbium^29,38,39^ based mode-locked fibre lasers, only selective studies have been conducted in thulium (Tm) fibre lasers^40^ so far. Since Tm based laser fibres provide a broad emission wavelength in the eye-safe region, from 1.7 μm to 2.1 μm, they have become popular for numerous applications including LIDAR, remote sensing, optical metrology, nonlinear mixing^41^ and biomedical surgeries and treatment^42,43^. Thus, our focus in this paper is to study induced chaotic states in a thulium fibre laser system. Since in previous studies ring cavity configurations were exclusively investigated regarding extreme events in mode-locked fibre lasers, the repetition rates usually hovered around the order of tens of MHz. In this study, for the first time, we present the generation of extreme optical pulsation in a mode-locked linear cavity laser at a higher repetition rate of 135 MHz. This is a factor of ~7.9 higher than in previously demonstrated ring cavity lasers with repetition rates up to 17.2 MHz^21^, which were designed to increase the effect of the weak nonlinear attractors. The optical extreme events can be partially attributed to modulation instabilities caused by the excessive intracavity power that does not contribute to the ultrafast pulse formation due to the gain and soliton pulse dynamics. The presented laser cavity includes a longer gain segment, so that overall reabsorption of the laser light is more pronounced, resembling a more dissipative system. The level of extreme behavior is controlled by the manipulation of the net cavity birefringence. Further, the ultrafast pulses in this transient chaotic regime do not feature any soliton condensation (i.e. bunching, explosions or collisions) nor any noise like pulsing. Instead, the conventional solitonic pulse shape is preserved while additional energy is being shed in a chaotic manner. The level of the chaos (the occurrence of the extreme events) is reproducibly controlled by employing an inline polarization controller in the fibre laser cavity. In order to further understand the effect of the molecular laser transitions in the extreme event generation process, the noise characterization of the optical extreme states in a mode-locked fibre laser is performed in great detail. This extreme optical pulsation regime also shows resemblances with rogue wave behaviour based on a statistical analysis. We believe that the generation of controllable chaotic states in a compact Tm based mode-locked fibre laser can pave the way towards the design of novel laser systems for burst pulse applications and can improve laser noise and stability operating regimes based on new fundamental insights into the cavity dynamics. Moreover, the optical system can serve as a testbed for controlled generation of chaos and can thus contribute to fundamental insights into the intricate interplay of nonlinear dynamics of rogue wave generation not only in optical systems but also for ocean waves. Further, this unique mechanism offers a wide variety of applications that benefit from high peak intensity pulses, including burst pulse amplification and micromachining. Also, the controllable degree of instabilities and randomness of the pulse intensities can pave the way for novel optical cryptography applications.

## Experimental set-up

**Figure 1:**
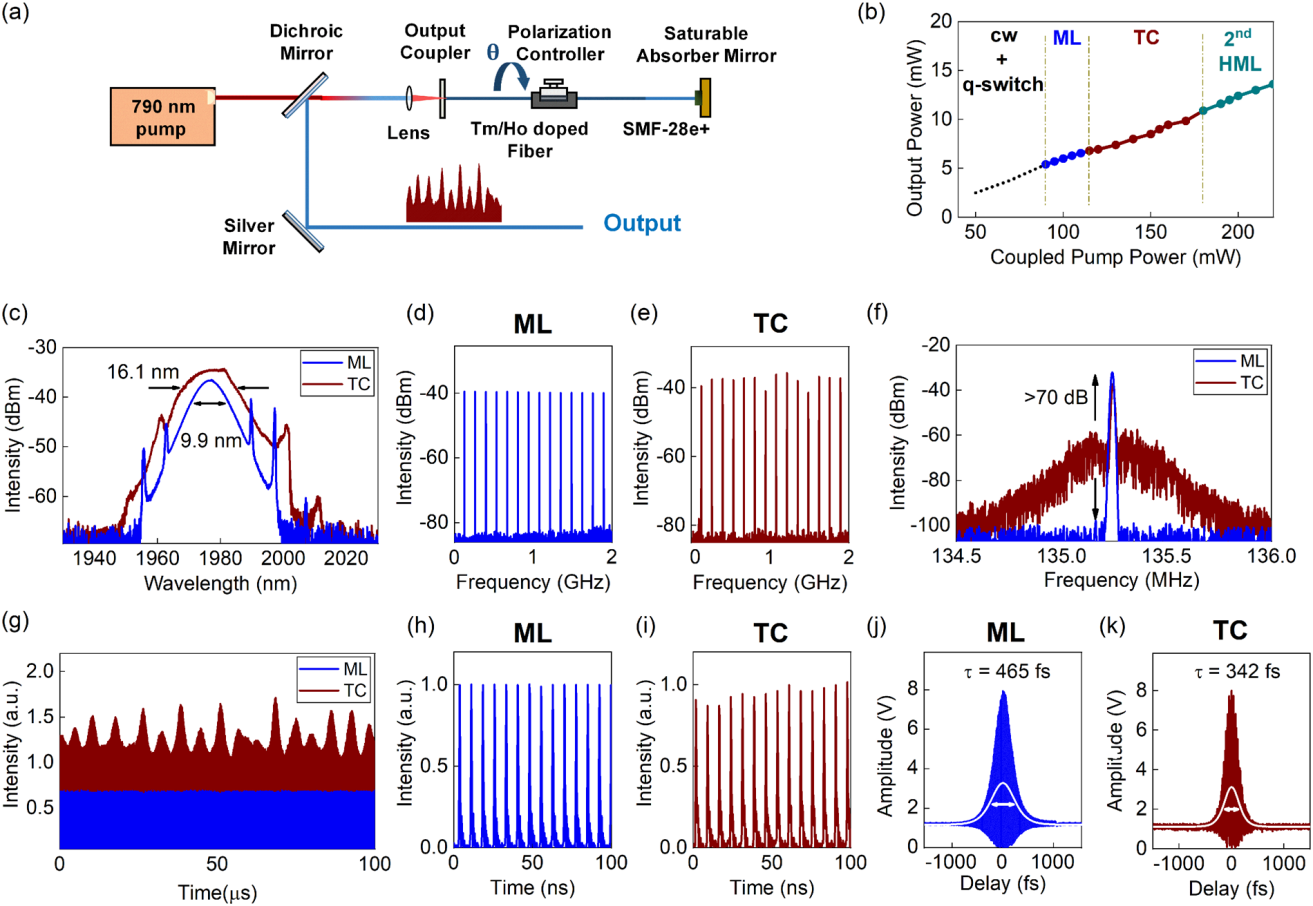
(**a**) Tm/Ho doped soliton mode-locked linear cavity fibre laser configuration. The cavity birefringence is manipulated with an inline polarization controller. (**b**) Different operating regimes are induced in the laser cavity with respect to the coupled pump power levels, where the transient chaotic (TC) state is compared with single pulse mode-locking (ML). The optical spectrum (**c**) for the TC state features a wider spectral bandwidth, whereas the wide band RF spectrum (**d**–**e**) clearly shows intensity fluctuations for the TC state (**e**). The RF spectrum of the fundamental repetition rate at 135.2 MHz shows a high contrast of >70 dB (**f**), while the long range oscilloscope traces (offset in intensity) illustrate the amplitude modulation for the TC regime (**g**). The single pulsing behaviour is confirmed in short range oscilloscope traces (**h**–**i**), and the interferometric autocorrelation traces (**j**–**k**) demonstrate shorter pulse durations for the TC state.

In order to achieve transitional chaotic states (TCs), a 75 cm long fibre laser cavity is constructed, as illustrated in Fig. 1(a). The linear fibre laser cavity includes a Tm/Ho doped silica based single-clad fibre (70 cm TH512, from Coractive) as the gain medium. A passive fibre segment (5 cm SMF-28e+) is spliced to the gain segment that is coupled to the saturable Bragg reflector (SBR). Self-starting mode-locked (ML) operation is induced by the SBR. The SBR (SAM-2000-20, from Batop GmbH) features a peak saturable modulation at a centre wavelength of 1960 nm with a relaxation time of 10 ps. The selected SBR has relatively high saturation fluence of 65 μJ/cm^2^ and a modulation depth of 12%. These values can support more pronounced intensity fluctuations in the fibre laser cavity. The fibre cavity is optically pumped at the peak absorption wavelength of the gain fibre, corresponding to a wavelength of 790 nm. The pump light is directly focused into the gain fibre core through an aspheric lens. An output coupler (OC) is coupled to the gain fibre segment of the cavity for an output coupling ratio around 10%. An external dichroic mirror (DM) serves to separate the different wavelengths of the pump and laser light. In order to control the net cavity birefringence, an inline polarization controller (PC) is utilized on the gain fibre segment of the cavity. The conventional solitonic ML operation is ensured by a net anomalous group velocity dispersion of −0.11 ps^2^ in the cavity.

## Characterization of the mode-locked and transitional chaotic states

The ultrafast laser operates in different regimes depending on the coupled pump power: The fibre laser features an initial cw lasing threshold of 21 mW. The fibre laser operates in cw and q-switched pulsing states for coupled pump power levels below the ML operation threshold of 90 mW. For these states, the laser experiences either a noisy continuous or irregular longer pulse duration output at repetition rates lower than the corresponding cavity round trip time. Stable single-pulsing ML operation is reproducibly achieved in a self-starting manner for pump power values above the ML threshold until the coupled pump power exceeds 115 mW. However, a multi-pulsing state with equidistant temporal pulse spacing corresponding to a harmonic mode-locking (HML) regime is not observed until the coupled pump power levels are increased to 180 mW where the output power is approximately twice the ML threshold. This agrees with prior recordings^44^,where the generation of transitional states between single- and multi-pulsing regimes has been observed. Between the single-pulsing and harmonically ML states, the laser switches into an intermediate state, which will be referred to as a transitional chaotic (TC) state for this study. The TC regime can be interpreted as a single-pulsing ML state with strongly pronounced amplitude fluctuations, despite its average output power showing an almost linear growth with respect to the increase in the coupled pump values. The progression of the average laser output power with respect to the coupled pump power levels is shown in Fig. 1(b).

The evolution of the optical spectrum from the single-pulsing ML to TC states is shown in Fig. 1(c). The ML state is studied for a coupled pump power of 105 mW providing an average output power of 6.8 mW. The optical spectrum of the ML state has a peak emission wavelength of 1970 nm with a full width half maximum (FWHM) of 9.9 nm, corresponding to a transform-limited pulse duration of 410 fs. The conventional solitonic sech shape optical spectrum is maintained until the laser switches into the TC state. The TC state features a much broader optical spectrum with a wider FWHM of 16.1 nm and an output power of 8.5 mW, when pumped with a coupled power level of 150 mW, which implies shorter pulse durations. The optical spectrum of the TC state continues to resemble most closely a sech shape, as the profile is wider than a Gaussian and narrower than a parabola. Both the ML and TC states feature characteristic Kelly sidebands due to periodic perturbations.

The wide band RF spectral comparison of the ML and TC states is shown in Fig. 1(d,e). While the ML state features a conventional uniform and equal intensity distribution of the higher RF harmonics, the RF spectrum of the TC state is characterized by intensity fluctuations on top of the conventional RF peaks. These fluctuations imply an overall intensity fluctuation is experienced by the ultrafast pulse train in the TC state. The analysis of the RF trace of the fundamental repetition rate at 135.2 MHz, matching the 75 cm long cavity, as shown in Fig. 1(f), features a marked difference between both states: the TC state is characterized by a wide pedestal around the RF peak, while the fundamental RF trace of the ML state has a narrow-band single peak with a signal to background ratio greater than 70 dB (for measurements with a RBW of 5 kHz). The lower signal to background ratio of ~35 dB confirms that mechanisms similar to the traditional formation of ultrafast pulsation are relevant for the TC state, while the wide pedestal indicates enhanced noise and increased pulse-to-pulse jitter.

Long range temporal characteristics of the ML and TC states are shown in Fig. 1(g) for a time span of 100 µs, including more than 13,500 femtosecond pulses. The ML state is characterized by a uniform and equal intensity distribution of the ultrafast pulses with a periodicity of 7.4 ns, agreeing well with the fundamental repetition rate. The TC state still maintains the single-pulsing formation, though strong overlaid intensity fluctuation are clearly visible. This is confirmed with oscilloscope traces for the ML and TC states for a short time interval of 100 ns, as shown in Fig. 1(h,i). The effect of photodetector ringing can be seen in these single-pulsing regime oscilloscope traces, yielding single-sided wings, while a strong amplitude modulation in the TC state is presented.

The single pulse temporal characteristics of the ML and TC states are measured in a custom-built interferometric autocorrelation (IAC) configuration, cf. Fig. 1(j,k). The ML state has an IAC trace yielding 465 fs long sech pulses with a minimum to maximum intensity ratio of 1/8. The TC state features a short pulse duration of 342 fs, while intensity fluctuations slightly deteriorate the average autocorrelation response. The IAC trace for the TC state does not show any pedestals nor wings, which rules out any formation of noise-like pulses or soliton condensation (i.e. in the form of soliton bunching, molecules or explosions). Also, the IAC features a similar intensity profile to the ML state which is broader than the corresponding IAC trace of a Gaussian pulse, indicating that the pulse profile does not deviate significantly from the sech shape.

## Route to extreme optical pulsation

The proposed fibre laser configuration has an energy gap where multi-pulsing states are not supported when the pump power is increased beyond the upper limit of the single-pulsing ML operation. The lower and the upper bounds of the supported pulse energy levels in the laser cavity are dictated by the saturation threshold of the SBR and the combined gain emission bandwidth of the cavity elements, respectively. Unique to the studied configuration, the single-pulsing regime does not extend up to a level where sufficient intracavity pulse energy is accumulated for pulse break up into two pulses with energies corresponding to the ML threshold. This is shown in Fig. 1(b), which indicates that a minimum of 90 mW of coupled pump power is required to achieve ultrafast pulse formation whereas the allowed upper limit for steady-state solitons within the cavity is 115 mW. Consequently, a harmonically mode-locked multi-pulsing state that is governed by soliton energy quantization does not exist for pump power levels between 115 mW and 180 mW. Thus, TCs states are observed between the single-pulsing and multi-pulsing ML regimes as an intermediate regime.

These TC states feature excessive intracavity energy beyond the soliton formation energy, which results in intensity fluctuations superimposed on the pulse train, cf. Fig. 1(g). Since this additional energy is shed in an irregular manner and is coupled to either a cw wave or pulsation resembling q-switching, significant increase in the pulse train instability is expected.

**Figure 2:**
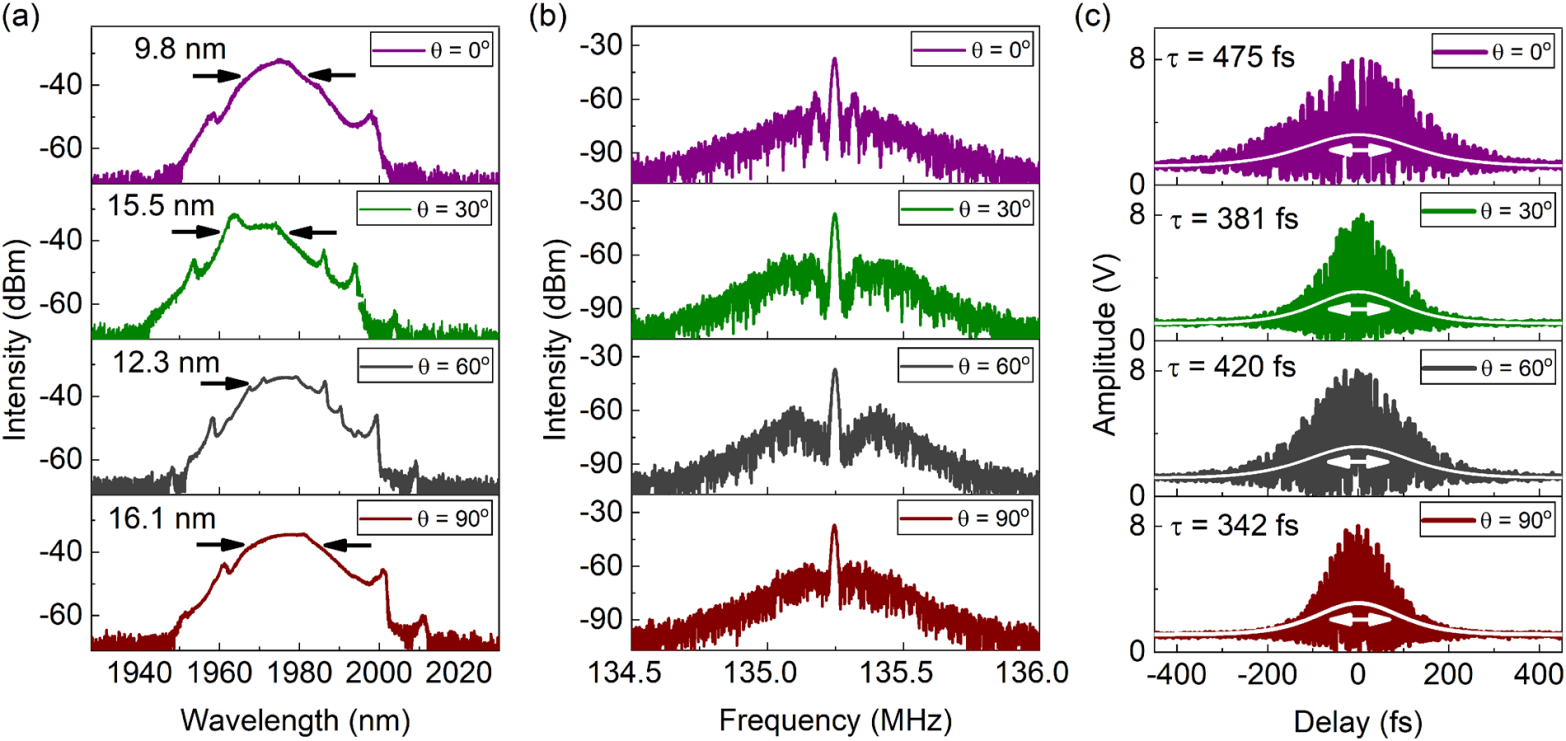
(**a**) The optical spectral bandwidth and shape in the TC state can be directly influenced by the intracavity polarization controller setting. (**b**) RF spectrum of the fundamental repetition rate shows slightly modified enhancement of RF wings. (**c**) Interferometric autocorrelation traces for the TC states under different net cavity birefringence at PC angles of 0°, 30°, 60° and 90°.

Since the energy gap sets a global limit, it is predicted that the net cavity birefringence influences the TC states without any formation of multi-pulsing states. To investigate the dynamics of TC states under different birefringence levels, an inline PC is incorporated on the gain fibre segment. The optical, RF spectral and temporal properties of the ultrafast pulse trains are shown in Fig. 2. In Fig. 2(a), the evolution in the optical spectrum of the TC states for different angle adjustments *θ* of the inline PC is demonstrated. The optical spectral shape and bandwidth are impacted by the PC angles, while the location of the sidebands remains fairly constant. The overall intensity profile is still best described by a sech shape (i.e. wider than a Gaussian and narrower than a parabola). This indicates that the excess pump power does not have a significant effect on the intrapulse shaping mechanism. Also, the overall spectral emission bandwidth is enhanced beyond the soliton spectral bandwidth of the ML state. The spectral FWHM varies between 9.8 nm up to a maximum value of 16.1 nm for a PC angle of 90**°**. All these TC states feature almost similar output average power levels, varying between 8.3 mW and 8.6 mW. Additional emerging structures on the optical spectrum, which diverge from the conventional sech intensity profile, also point towards the irregularity of the generated pulse trains. The RF spectrum of the fundamental repetition rate is studied in Fig. 2(b), showing the formation of a pedestal, implying an increase in the ultrafast pulse train instability (RBW of 5 kHz). In Fig. 2(c), the IAC autocorrelation traces illustrate the formation of ultrafast pulses with temporal durations shorter than the ML state. Femtosecond pulses as short as 342 fs are achieved, which are shorter than the pulse durations achieved in the single-pulsing ML regime. However, the average response from the autocorrelation of multiple pulses shows a noisier temporal profile for the femtosecond pulses, confirming the increased instability in the pulse train and the amplitude fluctuations. None of the IAC traces deviate from the sech pulse profile, confirming the dominant intrapulse shaping mechanism is not manipulated by the excessive pump power. As the IAC traces do not feature any pedestals or wings, single soliton pulse formation as the fundamental pulse shaping mechanism is supported.

**Figure 3:**
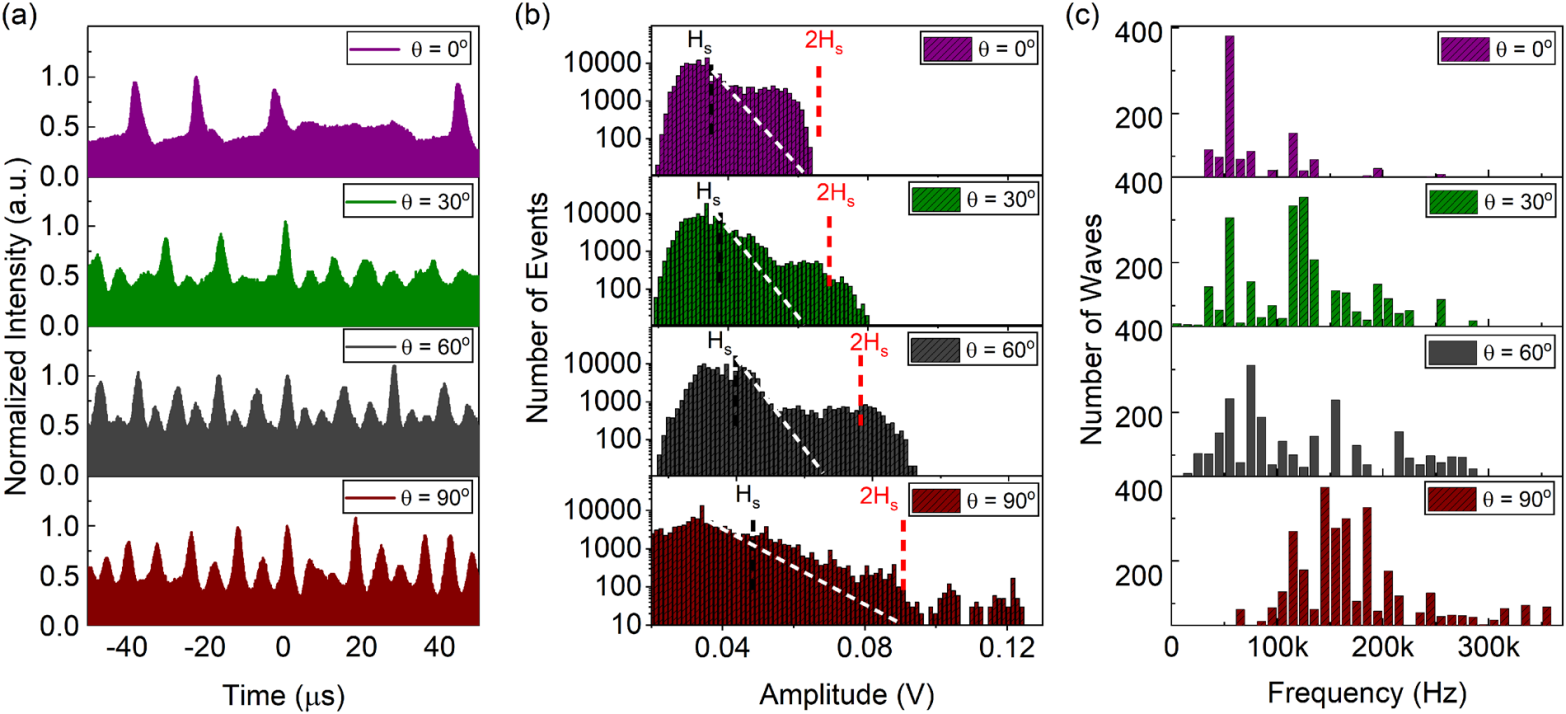
(**a**) The evolution of the long range oscilloscope traces of the TC states for different net cavity birefringence settings. The extreme events occur more frequently for larger PC angles. (**b**) Distributions of the pulse energies in the TC states. For PC angles higher than 30°, the TC states demonstrate stronger extreme behaviour due to formation of pulses with energies exceeding twice the significant wave height *H*_*s*_, which is characteristic for rogue waves. (**c**) The distribution of the frequency components of the intensity fluctuations indicate a partially split distribution for more frequently occurring extreme events.

To quantify the increased intensity fluctuations, a detailed characterization for the long range temporal domain, as well as the statistics of the fluctuation behaviour is investigated in Fig. 3. The changes in the intensity fluctuations for a temporal window of 100 µs are shown in Fig. 3(a). Both the shape and frequencies of the optical waves undergo a significant change with respect to the PC angle. Further, with a larger PC angle, it is observed that the modulation depth and the temporal quasi-periodicity is enhanced. This indicates that the number of unexpected (or explosive) events also increases, which in turn leads to the generation of more chaotic ultrafast pulse trains. As discussed in other rogue wave studies^45^, the generated extreme pulsation differs from conventional q-switching behaviour since the overall waves do not repeat themselves regularly but appear abruptly and disappear immediately.

Therefore, the statistical analysis of the number of events with respect to the pulse intensities is studied in detail. In Fig. 3(b), the occurrence of explosive events is shown and evaluated based on a conventional statistical analysis in histogram plots, accounting for more than 2∙10^6^ ultrafast pulses. The histogram plots feature a clear deviation from the classical distribution (shown in white dashed lines) with higher amplitude and frequency distributions, indicating the formation of extreme events. The classical distribution deviation is achieved by a fit of the first linear regime after the peak amount of events in the histograms. Also, the histograms indicate a distribution resembling to rogue wave behaviour due to the longer tail of higher amplitudes, in particular for large PC angle settings close to 90°. Increasing the PC angle, it is observed that the significant height (*H*_*s*_, the average amplitude of the highest one third of the pulses in the ultrafast train) is surpassed by at least two times for the PC angles higher than 30**°**. The existence of pulse amplitudes exceeding twice *H*_*s*_ is the conventional figure of merit in defining the rogue behaviour for optical or ocean waves.

Therefore, the generated ultrafast pulse trains for PC angles of 30**°** and larger clearly indicate an increased level of chaos and amplitude fluctuations that resemble rogue wave behaviour. This is further supported by the RF domain statistics presented in Fig. 3(c), where the frequency constituents of each wave are decomposed into intensity fluctuations. While the generated waves exhibit a narrower frequency range for PC angles lower than 30**°**, the frequency bandwidth widens and partially splits for larger angles. The partially split frequency components of the waves correspond to an approximate frequency doubling scheme or bifurcation, which is another indicator for increased irregularities in nonlinear systems. Further, the broadened frequency constituents conform to the formation of the pedestal observed in the RF spectrum shown in Fig. 2(b).

## Noise characteristics of the extreme optical pulsation

**Figure 4:**
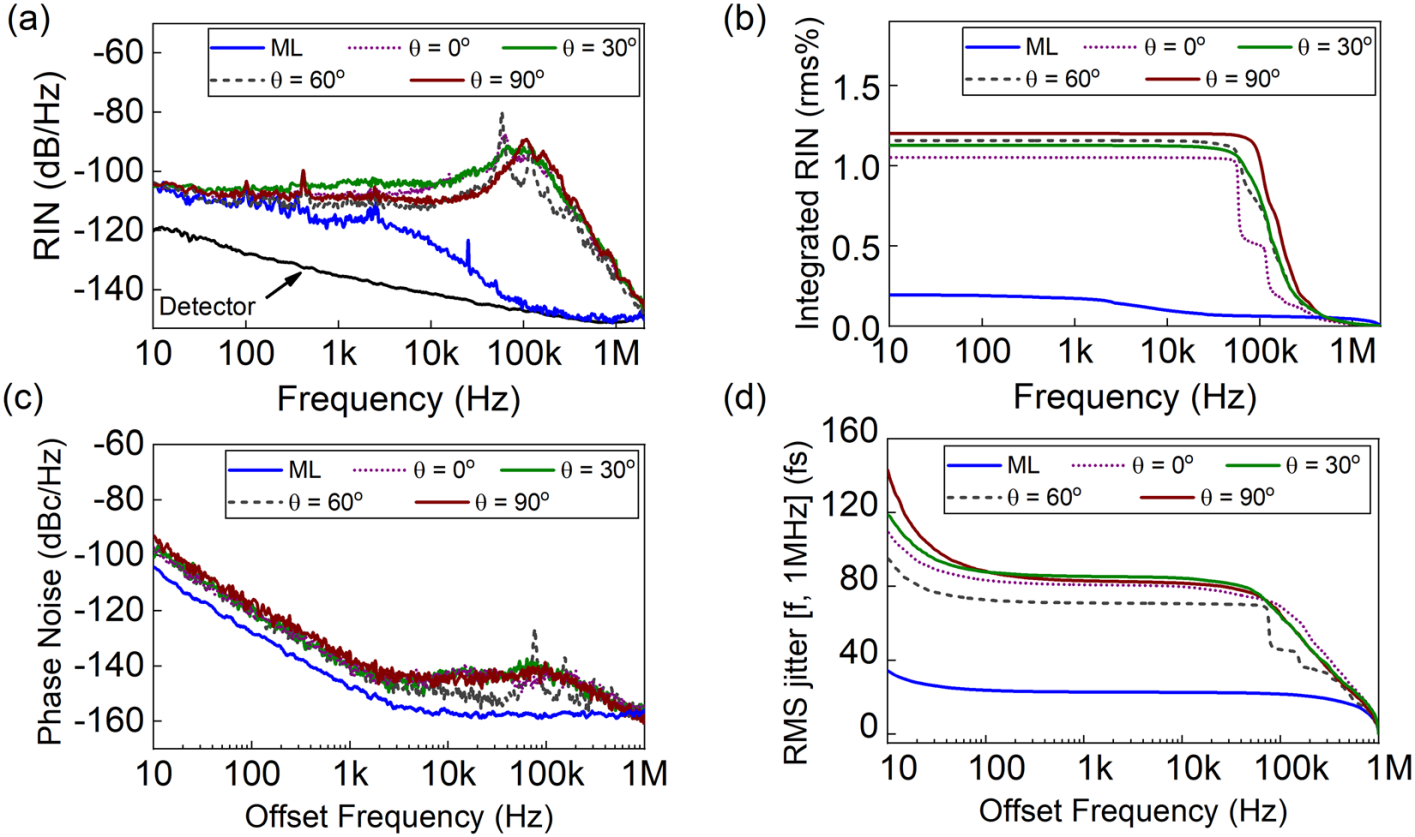
Noise characteristics of the ML state and TC states under different net cavity birefringence settings. (**a**) Relative intensity noise with significantly enhanced relaxation oscillations for the TC states. (**b**) Corresponding integrated rms intensity fluctuations. (**c**) Single side-band phase noise. (**d**) Corresponding pulse timing jitter. The elevated noise levels for the noise frequencies in the vicinity of 100 kHz implicate the influence of the relaxation oscillations in the formation of extreme pulsation.

Another important conclusion from the RF domain statistics of the intensity fluctuations is the overlap between the frequencies of the fluctuations and the relaxation oscillations of the Tm/Ho based gain media. Thus, a detailed investigation in the noise characteristics is conducted. Figure 4(a) shows relative intensity noise (RIN) measurements conducted for each TC state under different PC settings as well as for the ML state for the frequency interval of 10 Hz to 2 MHz. The RIN measurements are performed with a photodetector (PD-Thorlabs PDA10D) at optical power levels around 400 µW to maintain a PD response in the linear regime. With a corresponding shot noise level of −153 dB, the measured TC RIN curves are not limited by the detector or imstrument noise floor. The RIN noise curve indicates that relaxation oscillations are strongly pronounced for the TC states. The RIN curve features increased noise between 58 kHz and 162 kHz, peaking at 110 kHz, matching well the relaxation oscillation frequencies^46^ and the decay times of the excited state in Tm based gain media^47^. Further, the bifurcations in the wave frequency distribution shown in Fig. 3(c) are imprinted as peaks in the RIN curve for the TC states. The rms intensity fluctuations, cf. Fig. 4(b), reveal that the TCs states are one order of magnitude noisier than the ML state. However, the overall rms intensity fluctuations still remain below 1.3% for the TC states.

To further ensure long-term single-pulse formation (also confirmed by the interferometric autocorrelation measurements) under the strong intensity fluctuations, single side-band phase noise (PN) measurements are conducted with a photodetector (EOTech ET5000 with a 12.5 GHz bandwidth). The PN analysis for the TC states is compared with the ML state in Fig. 4(c). The TC states feature an increased PN level for the offset frequencies with respect to the fundamental repetition rate of 135.2 MHz in the vicinity of 100 kHz, matching the RF pedestals observed in Fig. 2(b). The TC states feature an increased level of pulse jitters as high as 95 fs over the interval of 10 Hz to 1 MHz, whereas the ML state has a jitter values of 25 fs as shown in Fig. 4(d). These measurements are conducted on a free-running laser. Thus, the low offset frequency noise can be further suppressed with additional stabilization and feedback circuitry. The overall pulse jitter values in TC states show that the long-term single-pulsing is well preserved, even though they exhibit more temporal noise than the ML state.

## Discussions and Conclusion

Chaotic optical pulsation with controllable levels of the occurrence of extreme events are demonstrated for the first time in a Tm/Ho doped soliton mode-locked linear cavity fibre laser. Unique to our observations, for the given cavity configuration, the laser enters into a transient chaotic pulsing regime for energies beyond the stability region of single soliton pulses that is characterized by an underlying single pulse formation at the fundamental repetition rate. Thus, extreme events occur due to excessive intracavity energy that is being shed in a disordered manner. This energy cannot contribute to the soliton formation anymore since the energy range is significantly below the harmonically mode-locking threshold. Instability regimes have been analysed based on energy rate equations depending on nonlinear gain dynamics^48^ and the existence of transition regimes with chaotic, periodic, noise-like and multi-pulsing behaviour has been previously demonstrated experimentally^44,49,50^. However, in contrast to the presented work in those systems, usually bifurcation behaviour as onset for multi-pulsing was recorded before chaotic operation was recorded. As the statistical analysis on these states has not been conducted in great detail so far, our studies offer new insights into this transient chaotic pulse operating regime. Similarly, previous studies on rogue wave and extreme event generation have been observed frequently through soliton condensation including intrapulse shape deviations from the conventional solitonic profile or through noise-like pulses. Thus, we show and analyse for the first time that extreme events can be obtained while a single solitonic pulsing structure is preserved. Further investigation of the exact pulse profiles and spectral build-up is ongoing to gain deeper insights into the intricate and complex dynamics. This study further represents the generation of extreme events in a relatively short linear cavity at a high repetition rate of 135 MHz compared to prior studies which have been exclusively conducted in ring laser cavities, with the highest repetition rate around 17.2 MHz^21^. While ring laser cavities offer a wide parameter space to manipulate, a linear laser cavity can be designed with low linear losses, thus allowing to study these effects at higher repetition rates. At the same time, the presented findings are different from conventional q-switching, where pulses with energy bursts usually occur regularly at repetition rates well below the fundamental repetition rate of the cavity. Although these are also characterized by statistics with high intensity frequency components^45^, the predictability based on regularly occurring events that can be anticipated sets that regime apart from classical rogue waves. The randomness of the fluctuations in the observed TC state in this paper indicates extreme behaviour, although the statistical histogram is more long-tailed compared to classical rogue wave distributions that rely on pulse collisions. Thus, our proposed configuration opens the pathway for deeper analysis of the underlying phenomena that lead to the occurrence of extreme events in a compact linear cavity fibre laser without requiring additional components providing artificial nonlinear attractors.

By changing the net cavity birefringence, the generation of the optical extreme events can be controlled. As the cavity does not feature any polarization selective component aside from the PC, nonlinear polarization dynamics and vector soliton generation are modified accordingly. Initial characterization measurements imply that the chaotic behaviour occurs in both orthogonal polarization eigenstates. Here, we focused on a detailed analysis of this transient chaotic state with an underlying single pulsing structure, as confirmed by the long-term temporal traces as well as the interferometric autocorrelation measurements. The deviation from conventional wave statistics confirms chaotic behaviour that can resemble rogue waves, depending on the net cavity birefringence.

Detailed intensity and phase noise performance of the extreme optical pulsations offers some insight into the underlying pulse shaping mechanisms and competing phenomena occurring in the TC state. The presence of enhanced noise can be related to relaxation oscillations and modulation instability, which can be enhanced in Tm gain fibres, and the interplay of reabsorption with the molecular laser level transitions. This indicates that the combination of the quality factor of the cavity and the gain fibre length exceeding its optical length for steady-state gain saturation can be considered interesting factors in the design of lasers with chaotic instabilities. As the instabilities were observed by incorporating a semiconductor saturable absorber with a higher modulation depth, detailed studies how the saturable absorber properties influence such transient pulse generation, also from two-dimensional materials or other saturable absorbers can be envisioned. Currently, for thulium fibre lasers, sufficiently high modulation depths seem to be advantageous for the observation of extreme events, since dissipative rogue waves were demonstrated with a MoS_2_ saturable absorber with a 14.6% modulation depth^40^.

We have presented a net anomalous based linear fibre laser cavity with a saturable absorber operating under fundamentally soliton mode-locking that can be pushed into a transient chaotic state based on the combination of pump power and intracavity birefringence settings. This marks a unique approach to generate extreme optical events whose statistical analysis indicates behaviour similar to rogue waves while showing distinct features that are different from previous studies. Thus, this novel method offers a rich analysis ground for generating extreme optical pulsation and can fuel a better understanding of the formation of chaotic ultrafast pulse trains. This effect is expected to be of general nature and not limited to the presented Tm/Ho fibre laser cavity as it can be translated to other fibre laser systems. By studying these instabilities that compete with stable cw mode-locking operation, knowledge into the contributing pulse shaping phenomena can be gained, enabling innovative cavity designs with improved performance and broader stability operating regimes. In addition, the chaotic pulse behaviour that is induced through control of the birefringence, supports extreme high peak intensities occurring with a certain irregular probability. These studies can enrich our insights into nonlinear dynamics and unique optical regimes that include characteristics from coherent but also stochastic states. As an optical testbed, they can elevate the understanding and predictability of extreme events and rogue waves in hydrodynamics, laser and supercontinuum optics or plasmas. This can advance laser cavity and nonlinear optical device designs and can be of interest for applications with high randomly distributed peak powers, as for burst pulse amplification, in micromachining or imaging. Further, it can boost applications where a degree of randomness and non-predictability is desired as for example in optical cryptography methods.

## References

[1] Fermann ME, Hartl I (2013). Ultrafast fibre lasers. Nat. Photonics.

[2] Okhotnikov O, Grudinin A, Pessa M (2004). Ultra-fast fibre laser systems based on SESAM technology: new horizons and applications. New J. Phys..

[3] Keller U (2003). Recent developments in compact ultrafast lasers. Nature.

[4] Collings BC (2000). Polarization-locked temporal vector solitons in a fiber laser: experiment. JOSA B.

[5] Cundiff ST, Collings BC, Bergman K (2000). Polarization locked vector solitons and axis instability in optical fiber. Chaos Interdiscip. J. Nonlinear Sci..

[6] Tang DY, Zhang H, Zhao LM, Wu X (2008). Observation of High-Order Polarization-Locked Vector Solitons in a Fiber Laser. Phys. Rev. Lett..

[7] Zhang H, Tang DY, Zhao LM, Tam HY (2008). Induced solitons formed by cross-polarization coupling in a birefringent cavity fiber laser. Opt. Lett..

[8] Kivshar YS, Turitsyn SK (1993). Vector dark solitons. Opt. Lett..

[9] Zhang H, Tang DY, Zhao LM, Wu X (2009). Observation of polarization domain wall solitons in weakly birefringent cavity fiber lasers. Phys. Rev. B.

[10] Lecaplain C, Grelu P, Wabnitz S (2014). Dynamics of the transition from polarization disorder to antiphase polarization domains in vector fiber lasers. Phys. Rev. A.

[11] Churkin DV (2015). Stochasticity, periodicity and localized light structures in partially mode-locked fibre lasers. Nat. Commun..

[12] Jeong Y, Vazquez-Zuniga LA, Lee S, Kwon Y (2014). On the formation of noise-like pulses in fiber ring cavity configurations. Opt. Fiber Technol..

[13] Horowitz M, Barad Y, Silberberg Y (1997). Noiselike pulses with a broadband spectrum generated from an erbium-doped fiber laser. Opt. Lett..

[14] North T, Rochette M (2013). Raman-induced noiselike pulses in a highly nonlinear and dispersive all-fiber ring laser. Opt. Lett..

[15] Akhmediev N (2016). Roadmap on optical rogue waves and extreme events. J. Opt..

[16] Qin H, Xiao X, Wang P, Yang C (2018). Observation of soliton molecules in a spatiotemporal mode-locked multimode fiber laser. Opt. Lett..

[17] Tsatourian V (2013). Polarisation Dynamics of Vector Soliton Molecules in Mode Locked Fibre Laser. Sci. Rep..

[18] Chouli S, Grelu P (2009). Rains of solitons in a fiber laser. Opt. Express.

[19] Bao C, Xiao X, Yang C (2013). Soliton rains in a normal dispersion fiber laser with dual-filter. Opt. Lett..

[20] Meng Y (2012). Multiple-soliton dynamic patterns in a graphene mode-locked fiber laser. Opt. Express.

[21] Lecaplain C, Grelu P, Soto-Crespo JM, Akhmediev N (2012). Dissipative Rogue Waves Generated by Chaotic Pulse Bunching in a Mode-Locked Laser. Phys. Rev. Lett..

[22] Runge AFJ, Broderick NGR, Erkintalo M (2015). Observation of soliton explosions in a passively mode-locked fiber laser. Optica.

[23] Renninger, W. H. & Wise, F. W. Dissipative Soliton Fiber Lasers. In (Wiley-Blackwell, 2012).

[24] Janssen PAEM (2003). Nonlinear Four-Wave Interactions and Freak Waves. J. Phys. Oceanogr..

[25] Akhmediev N, Ankiewicz A (2011). Modulation instability, Fermi-Pasta-Ulam recurrence, rogue waves, nonlinear phase shift, and exact solutions of the Ablowitz-Ladik equation. Phys. Rev. E.

[26] Kharif, C., Pelinovsky, E. N. & Slunyaev, A. *Rogue Waves in the Ocean*. (Springer-Verlag, 2009).

[27] Jumpertz L, Schires K, Carras M, Sciamanna M, Grillot F (2016). Chaotic light at mid-infrared wavelength. Light Sci. Appl..

[28] Peregrine DH (1983). Water waves, nonlinear Schrödinger equations and their solutions. ANZIAM J..

[29] Solli DR, Ropers C, Koonath P, Jalali B (2007). Optical rogue waves. Nature.

[30] Kibler B (2010). The Peregrine soliton in nonlinear fibre optics. Nat. Phys..

[31] Dudley JM, Genty G, Eggleton BJ (2008). Harnessing and control of optical rogue waves in supercontinuum generation. Opt. Express.

[32] Wabnitz S, Finot C, Fatome J, Millot G (2013). Shallow water rogue wavetrains in nonlinear optical fibers. Phys. Lett. A.

[33] Erkintalo M, Genty G, Dudley JM (2009). Rogue-wave-like characteristics in femtosecond supercontinuum generation. Opt. Lett..

[34] Montina A, Bortolozzo U, Residori S, Arecchi FT (2009). Non-Gaussian Statistics and Extreme Waves in a Nonlinear Optical Cavity. Phys. Rev. Lett..

[35] Finot C, Hammani K, Fatome J, Dudley JM, Millot G (2010). Selection of Extreme Events Generated in Raman Fiber Amplifiers Through Spectral Offset Filtering. IEEE J. Quantum Electron..

[36] Liu M, Luo A-P, Xu W-C, Luo Z-C (2016). Dissipative rogue waves induced by soliton explosions in an ultrafast fiber laser. Opt. Lett..

[37] Peng J, Tarasov N, Sugavanam S, Churkin D (2016). Rogue waves generation via nonlinear soliton collision in multiple-soliton state of a mode-locked fiber laser. Opt. Express.

[38] Liu Z, Zhang S, Wise FW (2015). Rogue waves in a normal-dispersion fiber laser. Opt. Lett..

[39] Dudley JM, Dias F, Erkintalo M, Genty G (2014). Instabilities, breathers and rogue waves in optics. Nat. Photonics.

[40] Wang P (2018). Dissipative Rogue Waves Among Noise-Like Pulses in a Tm Fiber Laser Mode Locked by a Monolayer MoS2 Saturable Absorber. IEEE J. Sel. Top. Quantum Electron..

[41] Sanchez D (2016). 7 μm, ultrafast, sub-millijoule-level mid-infrared optical parametric chirped pulse amplifier pumped at 2 μm. Optica.

[42] Jackson SD (2012). Towards high-power mid-infrared emission from a fibre laser. Nat. Photonics.

[43] Akosman AE, Sander MY (2017). Dual comb generation from a mode-locked fiber laser with orthogonally polarized interlaced pulses. Opt. Express.

[44] Bale BG, Kieu K, Kutz JN, Wise F (2009). Transition dynamics for multi-pulsing in mode-locked lasers. Opt. Express.

[45] Lecaplain C, Grelu P, Soto-Crespo JM, Akhmediev N (2013). Dissipative rogue wave generation in multiple-pulsing mode-locked fiber laser. J. Opt..

[46] Jackson SD, King TA (1999). Dynamics of the output of heavily Tm-doped double-clad silica fiber lasers. JOSA B.

[47] Moulton PF (2009). Tm-Doped Fiber Lasers: Fundamentals and Power Scaling. IEEE J. Sel. Top. Quantum Electron..

[48] Namiki S, Ippen EP, Haus HA, Yu CX (1997). Energy rate equations for mode-locked lasers. JOSA B.

[49] Smirnov S, Kobtsev S, Kukarin S, Ivanenko A (2012). Three key regimes of single pulse generation per round trip of all-normal-dispersion fiber lasers mode-locked with nonlinear polarization rotation. Opt. Express.

[50] Soto-Crespo JM, Grapinet M, Grelu P, Akhmediev N (2004). Bifurcations and multiple-period soliton pulsations in a passively mode-locked fiber laser. Phys. Rev. E.

